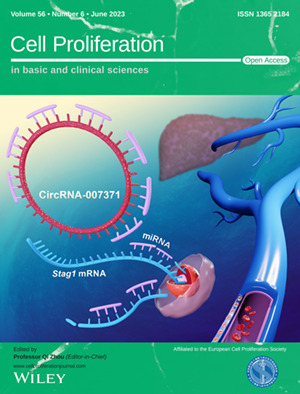# Featured Cover

**DOI:** 10.1111/cpr.13523

**Published:** 2023-06-20

**Authors:** Chong Zhao, Shuaijie Qian, Yang Tai, Yangkun Guo, Chengwei Tang, Zhiyin Huang, Jinhang Gao

## Abstract

The cover image is based on the Original Article *Proangiogenic role of circRNA‐007371 in liver fibrosis* by Chong Zhao et al., https://doi.org/10.1111/cpr.13432.